# Toxicity and Immunogenicity of a Tardigrade Cytosolic Abundant Heat Soluble Protein in Mice

**DOI:** 10.3389/fphar.2020.565969

**Published:** 2020-10-07

**Authors:** Harrison J. Esterly, Candice J. Crilly, Samantha Piszkiewicz, Dane J. Shovlin, Gary J. Pielak, Brooke E. Christian

**Affiliations:** ^1^ Department of Chemistry and Fermentation Sciences, Appalachian State University, Boone, NC, United States; ^2^ Department of Chemistry, University of North Carolina, Chapel Hill, NC, United States; ^3^ Department of Biochemistry and Biophysics, University of North Carolina, Chapel Hill, NC, United States; ^4^ Lineberger Comprehensive Cancer Center, University of North Carolina, Chapel Hill, NC, United States; ^5^ Integrative Program for Biological and Genome Sciences, University of North Carolina, Chapel Hill, NC, United States

**Keywords:** cytosolic-abundant heat-soluble proteins, immunogenicity, intrinsically disordered proteins, protein-based therapeutics, tardigrades, toxicity

## Abstract

Tardigrades are microscopic animals well-known for their stress tolerance, including the ability to survive desiccation. This survival requires cytosolic abundant heat soluble (CAHS) proteins. CAHS D protects enzymes from desiccation- and lyophilization-induced inactivation *in vitro* and has the potential to stabilize protein-based therapeutics, including vaccines. Here, we investigate whether purified recombinant CAHS D causes hemolysis or a toxic or immunogenic response following intraperitoneal injection in mice. CAHS D did not cause hemolysis, and all mice survived the 28-day monitoring period. The mice gained weight normally and developed anti-CAHS D antibodies but did not show upregulation of the inflammatory cytokines interleukin-6 and tumor necrosis factor alpha. In summary, CAHS D is not toxic and does not promote an inflammatory immune response in mice under the conditions used here, suggesting the reasonability of further study for use as stabilizers of protein-based therapeutics.

## Introduction

The emergence of the novel coronavirus SARS-CoV-2 has caused a global outbreak in coronavirus disease 2019 (Ahn et al., 2020). There is an acute need for a vaccine for this virus, and formulations are currently under development (Ahn et al., 2020; Shanmugaraj et al., 2020). However, once a vaccine is available, production challenges will be accompanied by difficulties in distribution, especially in developing countries. The preservation of protein structure and activity requires careful handling and low temperatures, which increases storage and transportation costs (Lee et al., 2017).

The 2018 Ebola outbreak in the Democratic Republic of the Congo illustrated the hurdles that must be overcome for refrigeration during storage, transport, and distribution of peptide-based therapeutics (Ashok et al., 2017). The Ebola vaccine must be stored at −60°C or below to prevent spoilage, and an emergency increase in cold chain capacity has been a priority since 2014 (Jusu et al., 2018).

In addition to vaccines, there are more than 200 FDA-approved protein- and peptide-based therapeutics on the market, including treatments for type 2 diabetes, breast cancer, and prostate cancer (Fosgerau and Hoffmann, 2015; Usmani et al., 2017). To stabilize proteins and increase shelf life, sugars, polymers, amino acids, globular proteins, and osmolytes have been investigated as excipients (Piszkiewicz and Pielak, 2019). Even in the presence of excipients, proteins must be kept at low temperatures. Lyophilization and desiccation can increase the shelf life of some proteins but may lead to problems such as aggregation and denaturation (Piszkiewicz and Pielak, 2019).

Efforts to stabilize proteins for dehydration and long-term storage at ambient temperatures have shown success with the tardigrade cytosolic-abundant heat soluble D (CAHS D) protein (Boothby and Pielak, 2017; Boothby et al., 2017; Piszkiewicz et al., 2019). CAHS D is intrinsically disordered, unique to tardigrades, and protects the activity of lipoprotein lipase and lactate dehydrogenase from desiccation, heat, and lyophilization (Piszkiewicz et al., 2019). The ability of CAHS D to protect both lipases and dehydrogenases suggests that the protection mechanism is generic and may be applicable to protein vaccine antigens such as hemagglutinin.

The application of CAHS proteins as excipients would be financially important and allow areas with little to no electricity infrastructure access to life-saving protein-based therapeutics. There are, however, a number of potential problems with using proteins from foreign organisms in therapeutic formulations, including allergic reactions and acute inflammation following administration.

Interleukin-6 (IL-6) and tumor necrosis factor alpha (TNFα) are inflammatory cytokines whose expression increases within 2 to 4 h following activation of the innate immune response (Papadakis and Targan, 2000; Zganiacz et al., 2004; Grivennikov et al., 2005; U.S. Department of Health and Human Services (DHHS), 2014; Francisco et al., 2015). IL-6 is produced in response to inflammation and stresses such as infection (Tanaka et al., 2014). Changes induced by IL-6 expression are often monitored in routine tests for inflammation and include increases in platelet release and induction of proteins such as C-reactive protein (Tanaka et al., 2014). TNFα is a pro-inflammatory cytokine secreted by macrophages that mediates acute inflammation (Chu, 2013). TNFα is also highly expressed in response to endotoxins. In contrast to the acute immune response, activation of the adaptive immune response is less immediate and involves the production of anti-drug antibodies that can interfere with drug action and reduce efficacy (De Groot and Scott, 2007; Baker et al., 2010). We injected CAHS D into mice to identify potential adverse responses, including weight loss and expression of IL-6 and TNFα.

## Materials and Methods

### Protein Purification

The expression and purification of CAHS D was carried out as described (Piszkiewicz et al., 2019). To summarize, the pET-28b plasmid encoding CAHS D (Boothby et al., 2017) was transformed into BL21 (DE3) *E. coli*. The bacteria were grown at 37°C until the optical density reached 0.6 at 600 nm. CAHS D expression was induced with 1 mM isopropyl-β-d-thiogalactoside (final concentration) for 3 h, after which cells were centrifuged at 4,500*g* for 25 min at 10°C. The supernatant was discarded, and the pellet was resuspended in 20 mM Tris pH 7.5. and stored at −20°C. Samples were thawed at 37°C and the cells were lysed by heat shock in boiling H_2_O for 15 min and then cooled to room temperature. Cooled suspensions were centrifuged at 15,000*g* for 45 min. The supernatant was diluted with an equal volume of buffer containing 8 M urea and 50 mM sodium acetate, pH 4.0, filtered through a 0.45 μm filter, and loaded onto a 5-ml FPLC cation exchange column (HiTrap SP HP, GE Healthcare). CAHS D was eluted over 29 column volumes with a salt gradient of 0 to 0.4 M NaCl in 8 M urea, 50 mM sodium acetate, pH 4.0. Fractions were analyzed by SDS-PAGE. Those containing pure CAHS D were pooled, transferred to 3,500 MWCO dialysis tubing (Fisher 68035), and dialyzed once against 20 mM Tris-HCl pH 7.5 for 4 h and then six additional times against deionized H_2_O. Purified CAHS D was once again sterile filtered through a 0.45-µm filter, flash frozen, and lyophilized. Lyophilized CAHS D was resuspended in endotoxin-free PBS to a concentration of ~20 g/L and then underwent two rounds of endotoxin removal by mixing with High Capacity Endotoxin Removal Resin (Pierce) overnight. The final endotoxin level was <10 EU/ml (<1 EU/injection) as quantified with the Chromogenic Endotoxin Quant Kit (Thermo Scientific Pierce). Purified CAHS D was analyzed by mass spectrometry, and a single peak was identified at m/z of 25353 Da (**Supplementary Figure 1**). The expected m/z is 25485.3 Da. The removal of the N-terminal methionine during expression in *E. coli* explains the difference between the expected and observed molecular mass (Wingfield, 2017). The final protein concentration was verified with a Bradford assay.

### Animals

C57BL/6 mice were purchased from Jackson Laboratory (Bar Harbor, Maine) and housed under standard laboratory conditions. Mice were bred and maintained in a temperature-controlled room with a 12-h light/dark cycle and provided standard chow and water *ad libitum*. Male and female mice were used indiscriminately. The welfare of all laboratory mice was monitored daily. All experiments were approved by the Appalachian State Institutional Animal Care and Use Committee.

### Statistical Analysis

Responses were compared using a homoscedastic two-tailed t-test (* indicates p ≤ 0.05, ** p ≤ 0.01, *** p ≤ 0.001, **** p ≤ 0.0001).

### Hemolysis

Blood from 3-month-old mice was collected by cardiac puncture under anesthesia with isoflurane and transferred to heparinized tubes (BD). Red blood cells from whole blood were pelleted at 1,000*g* for 10 min and washed twice with 10 times the pellet volume of Lactated Ringer’s solution (100 mM NaCl, 30 mM sodium lactate, 4 mM KCl, 1 mM CaCl_2_, VetOne). The red blood cell pellet was resuspended in Lactated Ringer’s solution to a final volume of 0.8% (v/v).

Equal volumes of Lactated Ringer’s solution, CAHS D (1.25 g/L, 2.5 g/L, 5 g/L, 10 g/L), or 1% Triton X-100 (MilliporeSigma) were mixed with the 0.8% red blood cell suspension for 30 min with rotation. The range of concentrations used was chosen based on the amount of CAHS D required to stabilize enzymes in vitro (Piszkiewicz et al., 2019). Lactated Ringer’s solution was used as a negative control because it is considered safe and causes minimal hemolysis (Ansel and Gigandet, 1976). Triton X-100 (1% weight/volume) was used as a positive control because it lyses red blood cells (Deibler et al., 1959). The suspensions were pelleted at 1,000*g* for 10 min, the pellets were discarded, and the absorbance of the supernatant was measured at 404 nm in the plate reader. The hemolysis experiment was performed in triplicate with blood from each of three mice. Comparisons between Lactated Ringer’s solution or CAHS D and Triton X-100 were performed.

### Toxicity of CAHS D and Anti-CAHS D Antibody Production

Mice were divided into four groups of six each and given 100 µL intraperitoneal injections at day 0 and day 21. The first group was given Lactated Ringer’s solution as a negative control. The next two groups were given low (1.25 g/L) or high (15 g/L) doses of CAHS D. The final group was given 0.05 g/L recombinant hemagglutinin protein (HA) from the influenza A H1N1 (A/Puerto Rico/8/1934) flu virus (Sino Biological 11684-V08H). The protein HA is often used in flu vaccines to induce anti-HA antibody production without toxicity (Henry et al., 2019). The HA injections were negative controls to show the specificity of the anti-CAHS D antibodies. All mice were monitored over a 28-day period for signs of toxicity, including weight loss, hunched posture, or changes in respiration. Blood was collected by submandibular vein puncture at day 0, 14, 21, and 28, and serum was purified with serum separator tubes (Sarstedt) and stored at −80°C. Antibodies against CAHS D were determined by ELISA assay as described below. The experiment was repeated twice more, for a total of 18 mice per treatment group. For anti-CAHS D antibody production and weight changes, values from 18 biological replicates of each CAHS D or HA sample were compared to PBS.

### Innate Immune Response

Mice were divided into seven groups of two to three each and given 100 µL intraperitoneal injections of Lactated Ringer’s solution, 1.25 g/L CAHS D, 2.5 g/L CAHS D, 5.0 g/L CAHS D, 10 g/L CAHS D, 15 g/L CAHS D, or 1 g/L lipopolysaccharides (LPS) from *E. coli* O127:B8 (MilliporeSigma L3129). Lactated Ringer’s solution was used as a negative control, and LPS was given as a positive control. LPS induces IL-6 and TNFα (Beurel and Jope, 2009). Four hours post injection, blood was collected by submandibular vein or cardiac puncture under anesthesia using isoflurane. Serum was purified with serum separator tubes and stored at −80°C. Serum was analyzed for IL-6 and TNFα by ELISA assay as described below. IL-6 and TNFα are markers of the proinflammatory cascade and indicators of inflammatory stress (Institute of Medicine (U.S.). Committee on Military Nutrition Research. et al., 1997). The experiment was repeated for a total of five to seven mice per treatment. Values for each assay were compared to values of the LPS positive control.

### ELISA Assay

To detect anti-CAHS D antibodies, each well in a MaxiSorp high binding ELISA plate (Invitrogen) was coated with 50 μL of 11 mg/L CAHS D in 0.1 M NaHCO_3_ buffer, pH 9.6, for 4 h and then blocked with 200 μL of 1% (v/v) casein (MilliporeSigma) overnight at 4°C. Mouse serum was diluted 1:200, and 50 μL of each sample was added to the plate in triplicate. The plate was incubated at 37°C for 1 h then washed four times with Dulbecco’s Phosphate Buffered Saline containing 1% (v/v) Tween 20 (PBS-T, MilliporeSigma). Donkey anti-mouse HRP antibody (GE Healthcare) was diluted 1:4000 and 50 μL was added to each well. The plate was incubated at 37°C for 30 min and washed four times with PBS-T. 1-Step™ Ultra 3,3′,5,5′-tetramethylbenzidine ELISA Substrate Solution (ThermoFisher Scientific) was warmed to room temperature and 50 μL was added to each well. The substrate was developed for 15 min in the dark and the reaction stopped with 50 μL of 2 M H_2_SO_4_. The absorbance was measured at 450 nm in the plate reader.

Levels of TNFα were quantified from serum samples with Mouse TNFα ELISA Kits (Boster and Invitrogen). Serum dilutions ranged from 1:5 to 1:10. The analytical sensitivity of the assay was 8 pg/ml (Invitrogen) and <1 pg/ml (Boster). Levels of IL-6 were quantified from serum samples with Mouse IL-6 ELISA Kits (Boster and Invitrogen). Serum dilutions ranged from 1:5 to 1:80. The analytical sensitivity of the assay was 4 pg/ml (Invitrogen) and <1 pg/ml (Boster).

## Results

### Hemolysis

**Figure 1:**
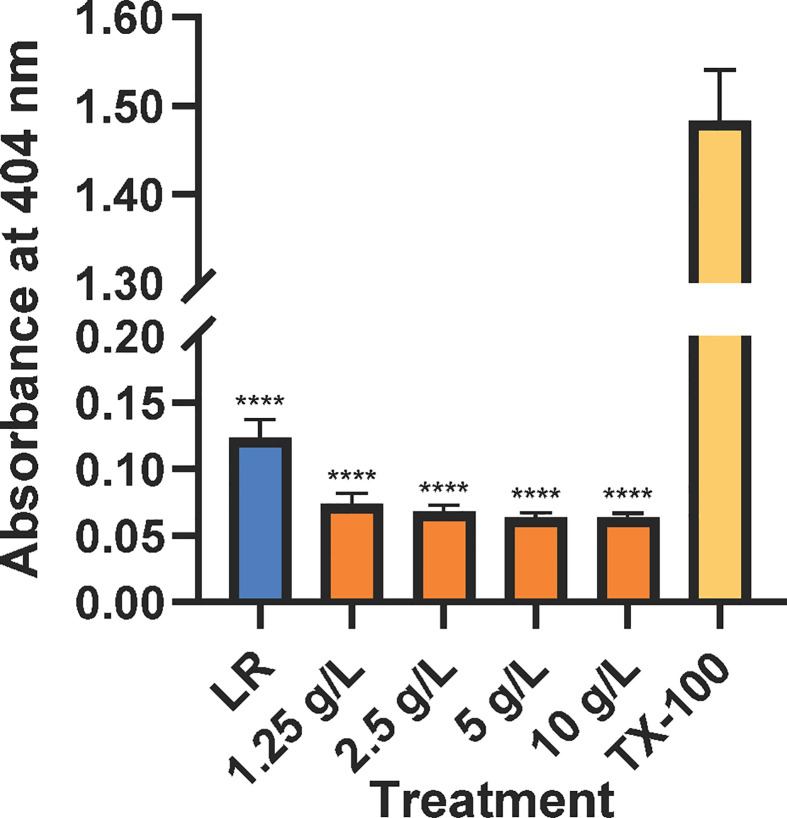
CAHS D does not causes hemolysis of mouse red blood cells. Lactated Ringer’s solution (LR, blue), CAHS D (1.25 - 10 g/L, orange), or 1 % Triton X-100 (yellow) was incubated with mouse red blood cells with rotation for 30 min. Intact red blood cells were pelleted, and released hemoglobin was measured at 404 nm. The hemolysis experiment was conducted using three technical replicates for each concentration. Additionally, the experiment was performed three times using blood from a new mouse each time. The technical replicates from each mouse were averaged. Error bars represent the standard error of the mean. Comparisons between Lactated Ringer’s solution or CAHS D and Triton X-100 were performed. **** indicates p ≤ 0.0001.

All CAHS D concentrations tested resulted in hemolysis at levels less than or similar to the negative control (**Figure 1**), Lactated Ringer’s solution. Lactated Ringer’s solution and all CAHS D concentrations elicited values less than the positive control, TX-100.

### Production of Anti-CAHS D Antibodies and Animal Health

**Figure 2:**
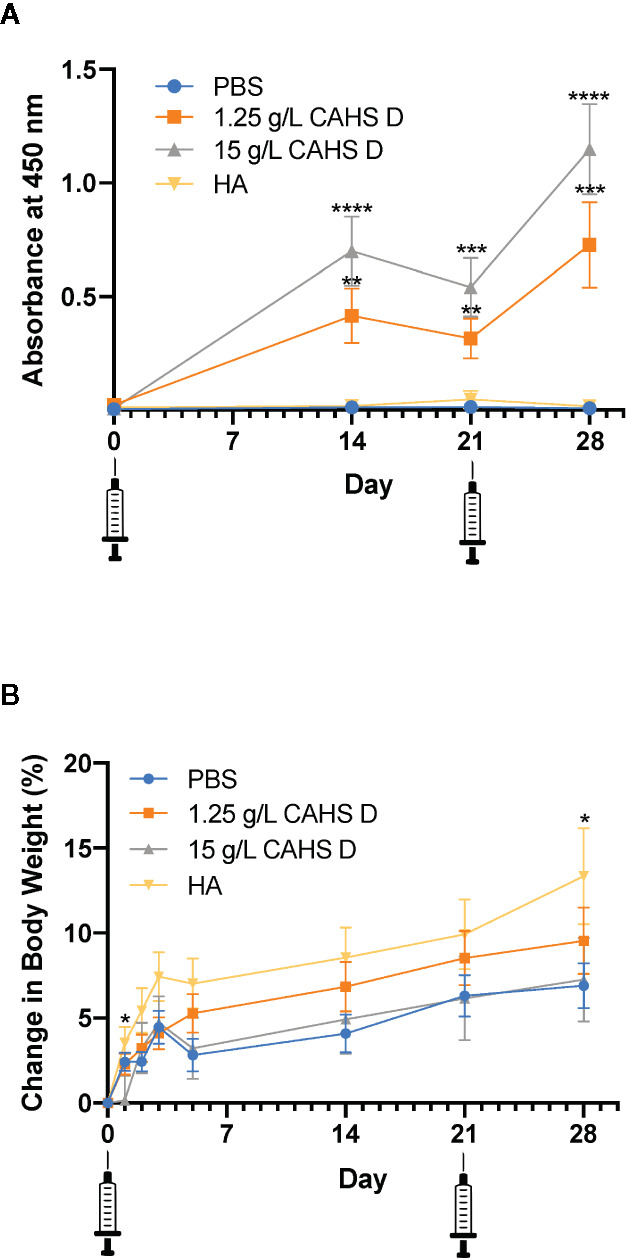
CAHS D injection produces anti-CAHS D antibodies but does not lead to weight loss. Anti-CAHS D antibody production **(A)** and weight changes **(B)** following CAHS D injection. Syringes indicate injection of CAHS D on day 0 and day 21. Mice were injected with 100 μL of phosphate buffered saline (PBS) buffer (blue), 1.25 g/L CAHS D (orange), 15 g/L CAHS D (gray), or 0.05 g/L hemagglutinin (HA) protein (yellow) from the H1N1 flu virus strain. Six mice were treated for each concentration, and the experiment was performed three times using a total of 18 mice per treatment. Each point represents the average of 18 data points. Error bars represent the standard error of the mean. For anti-CAHS D antibody production and weight changes, values from each CAHS D or HA sample were compared to PBS. At day 2 and 28, mice injected with HA showed more weight gain than mice injected with PBS. * indicates p ≤ 0.05, **p ≤ 0.01, ***p ≤ 0.001, ****p ≤ 0.0001.

Mice were injected with CAHS D (1.25 or 15 g/L) at day zero and given a second injection at day 21, designed to mimic a vaccine booster. Over the 28-day monitoring period, CAHS D at 1.25 g/L and 15 g/L was recognized by the mouse immune system following injection and elicited anti-CAHS D antibodies in serum at levels higher than PBS (**Figure 2A**). Anti-CAHS D antibody levels decreased at day 21, then increased following the booster injection. No significant difference in antibody levels was observed between the injection of 1.25 and 15 g/L CAHS D. No anti-CAHS D antibodies were produced in response to injection of buffer alone or the H1N1 hemagglutinin protein (HA), thus confirming the specificity of the ELISA assay (**Figure 2A**). No mice died during the study, had changes in ambulation, or lost weight due to CAHS D injection (**Figure 2B**).

### Production of Inflammatory Cytokines

**Figure 3:**
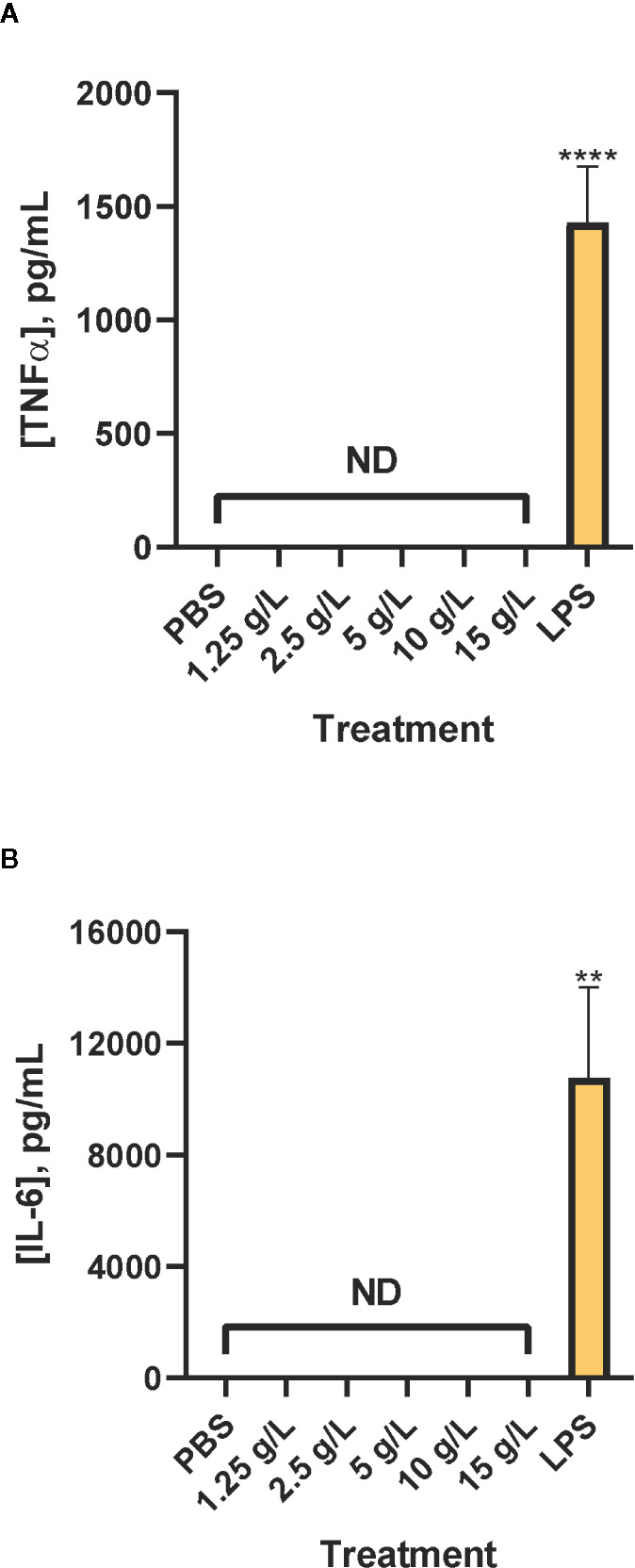
Inflammatory cytokines are not produced in response to CAHS D. Four hours after injection of PBS buffer (negative control), 1.25 - 15 g/L CAHS D or 1 g/L lipopolysaccharide (LPS, positive control), serum was analyzed for TNFα **(A)** and IL-6 **(B)**. Values less than the detection limit are labeled Not Detectable (ND). Five to seven mice were analyzed per treatment. In **(A)**, the bars represent the average of 6 replicates for PBS, 6 replicates each for 1.25 to 15 g/L CAHS D, and 7 replicates for LPS. In **(B)**, the bars represent the average of 6 replicates for PBS, 6 replicates each for 1.25 to 15 g/L CAHS D, and five replicates for LPS. In all panels the error bars represent the standard error of the mean. Values for each TNFα and IL-6 ELISA assay were compared to values of the LPS positive control. The values, except those from the LPS positive control, were less than the lower limit of quantification of the assay but were used to determine statistical significance. ** indicates p ≤ 0.01, ****p ≤ 0.0001.

Neither TNFα (**Figure 3A**) nor IL-6 (**Figure 3B**) were detected in levels above the limit of quantification. As a positive control, lipopolysaccharide (purified endotoxin) injection resulted in an inflammatory response that was higher than that elicited by PBS or CAHS D.

## Discussion

CAHS D caused less hemolysis than Lactated Ringer’s solution, which is known to be safe for intravenous injection (**Figure 1**) (Iqbal et al., 2018). Hemolysis of red blood cells can be dangerous and is induced by toxins such as those secreted from bacteria or present in snake venom (Condrea et al., 1964; Sogawa et al., 2018). The lack of hemolysis by CAHS D suggests it does not harm the structural integrity of red blood cells.

The production of dose dependent anti-CAHS D antibodies suggests that CAHS D was recognized by the mouse immune system following injection. Although antibody levels began to decline by 21 days post-injection, re-injection of CAHS D caused a second rise in antibody levels (**Figure 2A**). The development of anti-drug antibodies can interfere with the action of protein-based therapeutics but antibodies against CAHS D would not interfere with its intended purpose because CAHS D is not intended for use as a therapeutic; it would be used only to stabilize other therapeutic proteins during transport and storage, prior to injection. A BLASTp search of non-redundant protein sequences shows that CAHS D shares insignificant identity (<5%, E value <10) with any human or mouse protein, suggesting a low likelihood that antibodies to CAHS D will cross-react with endogenous proteins.

One measure of the toxicity of pharmaceuticals is whether they cause stress to animals. For example, the chemotherapeutic drug topotecan causes mice to lose as much as 30% of their body weight (Shah and Balthasar, 2014). In this study, mice were monitored for 28 days following injection of CAHS D and were re-injected at day 21. No mice died during the study, had changes in ambulation, or lost weight due to CAHS D injection (**Figure 2B**). All mice, including those injected with buffer alone, showed a 5% to 10% weight gain over a one-month period, which is typical for C57Bl/6J mice (Gargiulo et al., 2014) and consistent with changes in body weight with age for such mice maintained at The Jackson Laboratory, as stated in the Mouse Phenome Database, (http://www.jax.org/phenome). The typical weight gain and lack of obvious signs of stress suggest that CAHS D is safe for injection in mice.

The absence of TNFα and IL-6 induction following injection of CAHS D means that the mice did not initiate an inflammatory response to CAHS D and that efforts to remove endotoxins from the purified protein were successful. Taken together with the lack of hemolysis or weight loss induced by CAHS D, these results confirm that CAHS D does not illicit toxic or acute inflammatory immune responses in mice.

To be cost effective, the purification of CAHS D must be easy and have a high yield. One L of *Escherichia coli* expressing recombinant CAHS D can produce as much as 20 mg of purified CAHS D. However, removal of endotoxin is costly and time-consuming. It might be beneficial to optimize purification of CAHS D in insect cells or in endotoxin-free bacteria (Mamat et al., 2015; Planesse et al., 2015).

In conclusion, this study demonstrates that CAHS D does not cause hemolysis, notable distress, or an acute inflammatory response in mouse models. *In vitro* studies with enzymes indicate that lyophilization or desiccation with CAHS D can protect activity upon desiccation (Piszkiewicz et al., 2019). Future studies are needed to demonstrate that therapeutics stabilized with CAHS D remain effective following injection.

## Data Availability Statement

The raw data supporting the conclusions of this article will be made available by the authors, without undue reservation.

## Ethics Statement

The animal study was reviewed and approved by Applalachian State University Institutional Animal Care and Use Committee (IACUC).

## Author Contributions

HE and BC designed the study. HE, CC, SP, DS, and BC conducted experiments and analyses. HE wrote the first draft of the manuscript and prepared the figures. BC and GP obtained funding, contributed to study design and edited subsequent drafts. SP obtained funding, contributed to data interpretation and critically reviewed the manuscript. All authors contributed to the article and approved the submitted version.

## Funding

This work was supported by a University of North Carolina System Stage II Inter-Institutional Planning Grant (IPG) to BC and GP and the National Institutes of Health (R01GM127291 to GP). SP was partially supported by a dissertation completion fellowship from the UNC Graduate School. CC was partially supported by a fellowship from the National Science Foundation (DGE-1650116) and a NIH Training Grant (T32GM008570).

## Conflict of Interest

GP and SP are inventors on a patent application pertinent to this work (62/375,238).

The remaining authors declare that the research was conducted in the absence of any commercial or financial relationships that could be construed as a potential conflict of interest.
