# Supplementary material for: Toxicity and Immunogenicity of a Tardigrade Cytosolic Abundant Heat Soluble Protein in Mice
Source: Front Pharmacol. 2020 Oct 7;11:565969. doi: 10.3389/fphar.2020.565969 (PMC7577191; doi:10.3389/fphar.2020.565969)
Supplement: Supplementary file 1 [file Image_1.pdf]

Supplementary Material

1 Supplementary Figure

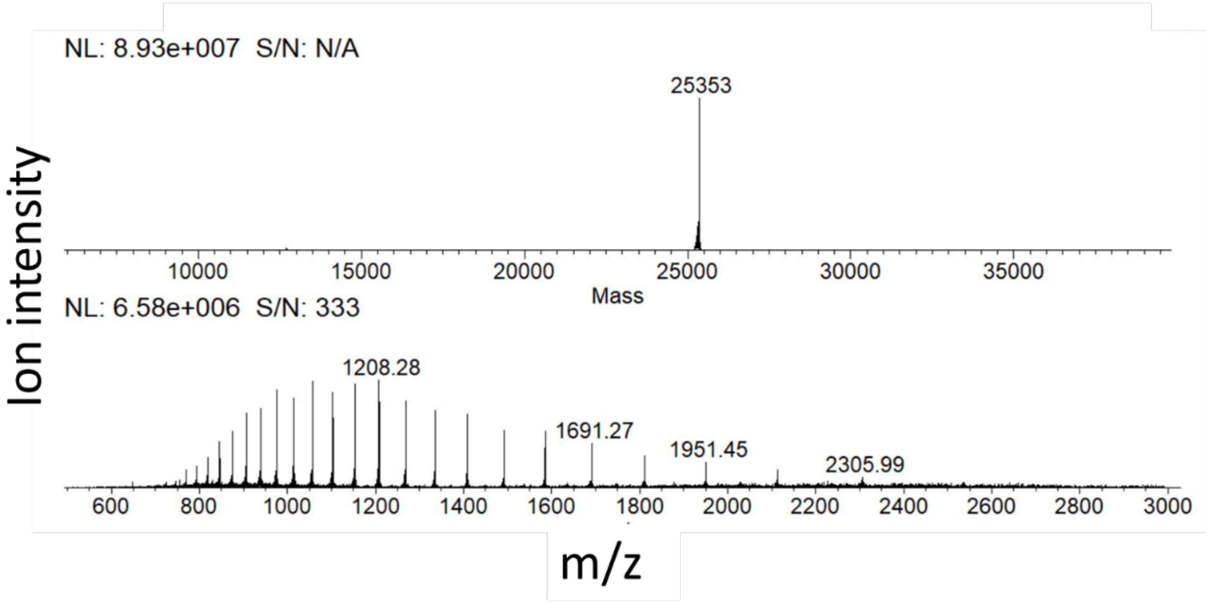

Supplementary Figure 1. Mass spectrum identifying a single peak at m/z of 25353 Da. The expected m/z is 25485.3 Da.
